# The impact of a local sugar sweetened beverage health promotion and price increase on sales in public leisure centre facilities

**DOI:** 10.1371/journal.pone.0194637

**Published:** 2018-05-30

**Authors:** Penny Breeze, Robert Womack, Robert Pryce, Alan Brennan, Elizabeth Goyder

**Affiliations:** 1 School for Health and Related Research, Sheffield, United Kingdom; 2 Sheffield City Trust, Sheffield, United Kingdom; University of California, San Diego, UNITED STATES

## Abstract

**Background:**

We aimed to evaluate the impact of a local sugar sweetened beverages (SSB) health promotion and 20p price increase in leisure centre venues and estimate the impact on consumption.

**Method:**

Monthly cold drinks sales data and attendance at leisure centres across the city of Sheffield were analysed over the period January 2015-July 2017. Interrupted time-series methods were employed to estimate changes in consumption per attendance of SSB and non-SSB cold drinks following the introduction of the SSB policy from August 2016 adjusting for seasonal variation and autocorrelation. SSB price elasticities were estimated with fixed effects log-log models by SSB product type (soda can, soda bottle, soda post mix, energy drinks, juice from concentrate).

**Findings:**

We estimated a 31% (95% CI 4%, 59%) reduction in units of SSB sold per attendance in the year since the policy was introduced. We did not observe substitution effects to fruit juice or water but found sales of other artificially sweetened non-SSB products increased by 27% (95% CI 6%, 47%) after the introduction of the tax. Price elasticity analysis identified that a 1% increase in price alongside health promotion leads to a 3.8% (95% CI 3.1% 4.4%) decrease in demand for SSB’s. Price elasticity of demand was highest for child friendly and high caffeine energy drinks.

**Interpretation:**

Demand for SSB drinks at leisure centre venues is highly responsive to the policy, particularly for child-friendly and high caffeine energy drinks, compared with other SSB tax policy evaluations. The policy also increased purchases of carbonated non-SSB.

## Introduction

The impact of obesity on associated chronic diseases has led to calls for a comprehensive public health approach to tackling the current trends in physical inactivity and high calorie diets. Leisure centres present an opportunity for broad health education and promotion influencing positive behaviour changes both in their service users and in their local communities. Public Health England (PHE)’s ‘Sugar reduction: the evidence for action’ report in 2015 specified eight areas for action in public areas, including leisure centres, to reduce sugar consumption, targeting price promotions, advertising, price manipulation, training and education.[1]

There is growing evidence that pricing policies for sugar sweetened beverages (SSBs) are effective in reducing the growing burden of obesity. A randomised controlled trial found that reductions in the consumption of sweetened drinks were associated with weight loss.[2] Additional evidence from prospective cohorts studies indicates an association between SSBs and type 2 diabetes.[3–4] An analysis based on data from the Global Burden of Disease study 2010 estimated a total of 184,000 deaths from diabetes (72%), CVD (24%), and cancers (4%) per year and 8.5 million DALYs attributable to SSB consumption.[5]

Several pricing policies for SSBs have demonstrated significant reduction in sugar drinks consumption. In Mexico a study on the effect on purchases of beverages from stores identified reductions in sales of SSBs across all income groups one year after implementation of the excise tax (approximately 10% increase) on SSBs.[6] In the UK a levy of £0.10 per drink on sales of SSBs introduced in a national chain of restaurants was evaluated and found to reduce sales of SSBs by 11.7% at 12 weeks.[7] A simulation study to appraise the health effect of industry responses to a soft drinks levy estimated a 32.7mL reduction in daily intake of SSBs following price rises, which corresponded to a 0.5% reduction in obesity, reduction in incidence of type 2 diabetes by 17.7 per 100,000 person years and reduction in incidence of dental caries by 2.4 per 1,000 person years.^8^ However, there is often scepticism regarding the effectiveness of national taxes on SSBs due to unpredictable responses by manufacturers and consumers. [8] In April 2018 a national levy on SSB will be introduced in the UK but there is uncertainty regarding the impact of the levy on sugar consumption. [9]

In July 2016 Sheffield City Trust (SCT) introduced a comprehensive sugar sweetened drink strategy to improve customer health outcomes at all of its Sheffield leisure facilities including:

1. £0.20 ($0.27, €0.23) increase on price of all drinks containing 5mg of sugar per 100ml or more directly applied to all products in leisure centre venues enforced by a central catering team. Approximately 11%-25% of product prices.
2. Staff training in order to deliver face to face awareness of scheme with customers
3. Publicity in local and national media
4. Publicity within venues including posters, billboards and drinks stickers in café and vending areas.

Details of the policy and its development can be found in S1 File.

Estimates from the NDNS suggest that 1.4% of all SSB purchases are made in leisure centre facilities.[10] Although the volumes SSB sales at leisure centres are small, leisure centres provide a unique context for delivering public health interventions. Venues implementing price rises have greater control over the impact on consumers and can enhance policies with health promotion and education. Furthermore, 16% of SSB’s are purchased outside of work or home indicating a wider health impact if pricing policies were extended to other public venues.[10]

The aim of this study is to appraise the impact of the pricing and promotional policies in leisure centres on consumption of SSBs. Prices of SSBs increased by 20p after July 2016 alongside health promotion initiatives, and these prices increases have been maintained throughout the study period. Using routinely collected sales data provided by SCT, we evaluated changes in the demand for cold beverages after the implementation of the pricing and promotional policy.

## Method

We obtained data on sales of cold drinks at all venues from January 2015 to July 2017. Cold drinks sales at cafes and vending machines within venues were included but sales data for hot drinks and other confectionary were not available. Each month the total numbers of cold drinks sold were extracted by product from routine monitoring of product sales used by the catering team for accounting and stock control purposes. Data for seven venues affected by the pricing policy and eight venues not affected by the policy were available. Each venue has a different mix of beverages on offer depending on the venue size and facilities. However, there were some commonalities across all venues. All venues sold carbonated soft drinks (cans, bottles, or post mix cups) with SSB and non-SSB options. All venues, except two golf courses, sold water. The most popular products were available throughout the study period, however the analysis includes some products that were available for limited periods.

The venues affected by the policy were public leisure centres providing a range of sporting facilities including swimming pools, gyms, ice skating, indoor athletics track, and other training facilities. The community leisure centres attract attendances from all age ranges, black and minority backgrounds and people with disabilities. The leisure centres are located across the city serving a diverse range of communities with high participation in lower sociodemographic areas. The venues vary in size and some include cafes and vending facilities, and some vending only. The venues not included in the policy included golf courses and entertainment venues. Data from the unaffected venues were extracted to describe patterns in sales in the absence of the policy over the study period. The population profiles attending each venue type are different so the unaffected venues do not represent a true control to the intervention, but are drawn from the same local population. It is useful to observe whether trends observed in the affected venues are also seen in the other venues to understand if changes were due to other external factors, such as media coverage of the national sugar drinks tax or other sugar-related local and national health promotion campaigns.

Over the same period monthly recorded attendances were extracted for each venue. We employed methods used by SCT to monitor visitors attending at classes, gyms, using sporting facilities, or attending events for routine accounts. Visitor numbers for some venues include some estimated values where exact numbers for some visitor types were not available (for example accompanying carers for children’s sports lessons or the number of attendees at a squash court booking). Data on product volumes and prices were obtained from the catering team. Attendance data were used to estimate average sales per attendance at the venues to account for variability in demand due to attendance volume. We obtained nutritional information for all beverages in the UK from publicly available sources to describe the sugar and calorie content of the products sold. [11]

### Statistical analysis

Differences in total sales before and after the promotions and pricing policy was introduced were assessed using two sample t-test. In addition two regression models were specified to describe changes in sales per attendance after the policy and price elasticity of demand in response to price changes. All statistical analyses were conducted using Stata 15. [12]

#### Interrupted time series analysis

It was not possible to construct a true experimental design to study the association between the SSB policy and purchases due to the timing of the evaluation after implementation of the policy. A control group was not planned in the development of the policy. Therefore, we applied an interrupted time series study design because the policy was introduced in a number of venues over a clearly defined time period. We conducted an interrupted time-series analysis using linear fixed effects models (xtreg) with venue as a fixed effect to assess whether there was a step-change in sales per visitor of levy-eligible SSBs in the period after implementation. Sensitivity analyses investigated the effects at individual venues, venues grouped by vending only and vending and café, using ordinary least squares regression. We also tested a sensitivity analysis using a mixed effects model to allow the intercept and slope to vary across venues. We grouped products into SSB, all non-SSB, carbonated non-SSB (excluding juice and water), non-SSB fruit juice and water. We also examined SSB by product type into groups of carbonated bottles (500ml), carbonated cans, carbonated post-mix (soda on draught), high sugar juice concentrate, and caffeinated energy drinks.

Model outcomes included all cold drink sales per attendance, SSB sales per attendance, non-SSB sales per attendance, non-SSB sales per attendance (excluding water and juice), non-SSB fruit juice, non-SSB water and volume of SSB sold per attendance. It was hypothesised that the intervention would have an immediate impact on sales on initiation of the policy and would maintain effectiveness over time. Investigations of the data suggested that sales per attendance were relative stable over the study period, i.e. no gradual increases or decreases over time and time trend covariates for month and year were not significantly associated with demand. Therefore, the model assumed no time trend in sales over time and a level change in SSB sales per visitor with no lag.

$$Y_{it}=\beta_{0}+\beta_{1}X_{t}$$

Where *Y*_*it*_ is the outcome at venue *i* at time *t*, *X*_*t*_ is a dummy variable indicating the pre-intervention period (coded 0), or the post-intervention period (coded 1). *β*_0_ represents the estimated baseline sales per attendee, and *β*_1_ is the level change following the intervention.

Average monthly rainfall was included to describe environmental factors. [13] Seasonality was adjusted for to account for seasonal changes in sales and because there was an unbalanced distribution of months before and after the intervention. We included two methods for adjusting for seasonal variations in sales. Firstly, to include a dummy variable for months with school holidays due to known increases in sales and attendance amongst families during school holidays. Secondly, to include Fourier terms to describe smooth fluctuations in sales. Fourier terms are pairs of sine and cosine functions describing a pattern covering the full calendar year. Fourier terms enable the model to capture peaks corresponding to school holiday periods, and were selected in our primary analysis due to superior model fit using the Akaike Information Criterion.

Serial correlation was tested using the Wooldridge test (xtserial command in STATA). The test did not identify serial correlation with the inclusion of Fourier seasonal adjustment terms and rainfall.

#### Price elasticity

Price elasticity measures the responsiveness of the quantity demanded of a good to a change in its price. Price elasticity is the ratio between the percentage change in the quantity demanded, *q*, and the corresponding percent change in price, *p*. We analysed the response of demand for all SSB’s at leisure venues to changes in prices and health promotion policy over the analysis period to generate generalisable estimates of the percent change in demand to a percent change in prices alongside health promotion. SSB drinks were disaggregated into five product types (can, bottle, post mix, energy drinks, high sugar juice from concentrate), and venue in a fixed effects linear model to account for heterogeneity in demand across venues and product types. Some products were not consistently available in any of the venues, due to bespoke orders for events. As a consequence, Schweppes, Lucozade, and Relentless were excluded from the analysis due to inconsistent availability (Less than 1% of all sales).

The log-log model to estimate the price elasticity of demand for SSB is described by,

$$log(q_{it})=\beta_{0}+\beta_{1}log(p_{it})+\beta_{2}v_{it}+(\beta X_{it})+\alpha_{i}+u_{it}$$

Where *q*_*it*_ is the quantity of sales for each product and venue, *i*, at each monthly time period of the analysis, *t*. *p*_*it*_ is the price for each product within venue, i, at each time period t. *v*_*it*_ is attendance for each venue by time periods *α*_*i*_ is the unobserved time-invariant individual effect for each product by venue, and *u*_*it*_ the random error term. Additional covariate adjustments were added to account for seasonal variations using Fourier terms, school holiday dummy variables and average rainfall as described above (***βX***_***it***_).

### Role of the funding source

The funders of the study had no role in study design, data collection, data analysis, data interpretation, or writing of the report. The corresponding author had full access to all the data in the study and had final responsibility for the decision to submit for publication.

## Results

The unadjusted mean total unit sales per month by venue and drink type are reported in Table 1 across the study period. SSB’s contributed over a third of total sales over the study period, with variation between venues with different types of facilities (over a half of soft drinks sales at the ice-skating facility “ICE Sheffield” were SSB’s). There was also substantial variability in the volume of sales across venues due to the differences in venue capacity and catering provisions. The analysis of all cold drinks sales per attendance showed a non-significant reduction in total monthly sales since the policy was introduced (Table 2). The change in sales per attendance of SSB since the tax was introduced is both large and statistically significant -0.016 (95% CI -0.011, -0.022). The increase in all non-SSB drinks was borderline significant 0.011 (95% CI -0.002, -0.020) and the increase in non-SSB excluding water and fruit juice was statistically significant, 0.009 (95% CI 0.004, 0.013). There were no significant changes in water or in fruit juice consumption since the tax was introduced.

**Table 1:** Total average unit sales per month by venue and drink type.

	All cold drinks		SSB		Non-SSB (inc. water + fruit juice)		Non-SSB (exc. Water + fruit juice)		Non-SSB Water		Non-SSB Fruit Juice	
Monthly sales	Mean	SD	Mean	SD	Mean	SD	Mean	SD	Mean	SD	Mean	SD
Hillsborough	4473	1120	1735	704	2738	575	1068	233	822	163	848	278
Ponds Forge	11511	2267	3666	1091	7845	1762	3142	934	3098	720	1605	521
Concorde	1697	544	460	231	1236	366	534	201	417	165	285	115
Springs	347	196	129	101	219	130	83	80	100	64	36	29
ICE Sheffield	9237	2322	4764	1389	4473	1167	2912	857	936	190	625	246
English Institute for Sport	5139	1965	1343	749	3796	1407	1610	551	1371	477	816	636
Heeley	252	147	56	43	197	126	64	60	77	45	56	48
All venues	32655	4575	12151	3151	20504	2948	9412	1619	6821	855	4271	1161

**Table 2:** Unadjusted difference before and after policy in unit sales per attendance per month by drink type.

	All cold drinks		SSB		Non-SSB (inc. water + fruit juice)		Non-SSB (exc. Water + fruit juice)		Non-SSB Water		Non-SSB Fruit Juice	
	Mean	St.error	Mean	St.error	Mean	St.error	Mean	St.error	Mean	St.error	Mean	St.error
Monthly sales per attendance before policy	0.113	0.004	0.049	0.002	0.065	0.002	0.029	0.001	0.022	0.001	0.014	0.001
Monthly sales per attendance after policy	0.108	0.005	0.032	0.002	0.076	0.004	0.037	0.002	0.024	0.001	0.014	0.001
Percentage change	-4.79%		-33.80%		16.97%		30.26%		7.77%		4.26%	
P-value	0.421		<0.0001		0.017		0.0003		0.256		0.702	

**Fig 1:**
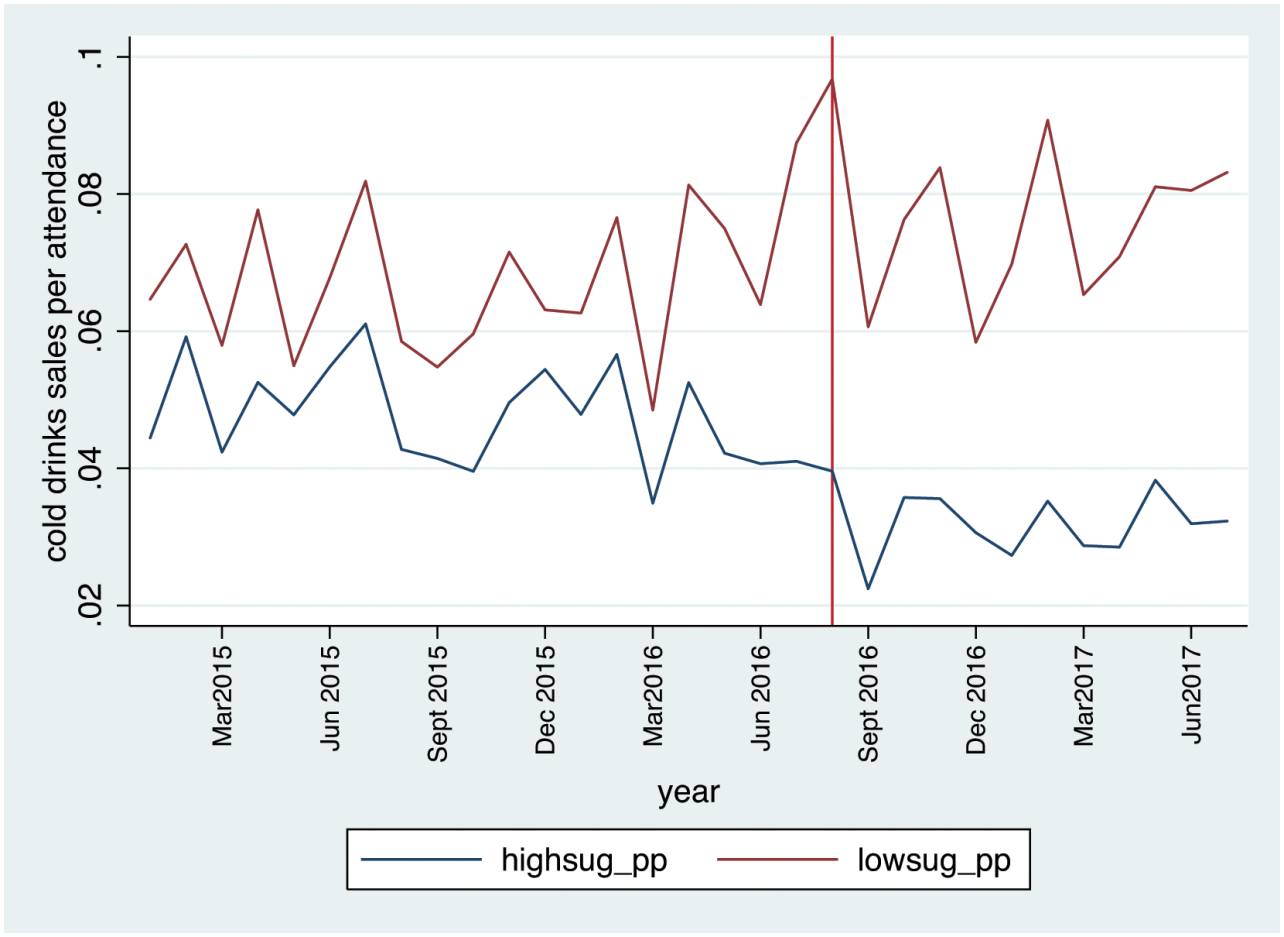
Monthly sales per attendee at affected venues for high and low sugar cold drinks during study period.

Fig 1 illustrates the monthly sales per attendance over the study period. The vertical red line illustrates the date that the policy was introduced. A time trend in sales per attendance was not observed in the pre-tax period. Although, monthly changes in sales per attendance are highly variable, the graph confirms that sales per attendance for SSB fall following the tax whilst the sales of non-SBB tend to be higher from August 2016.

**Fig 2:**
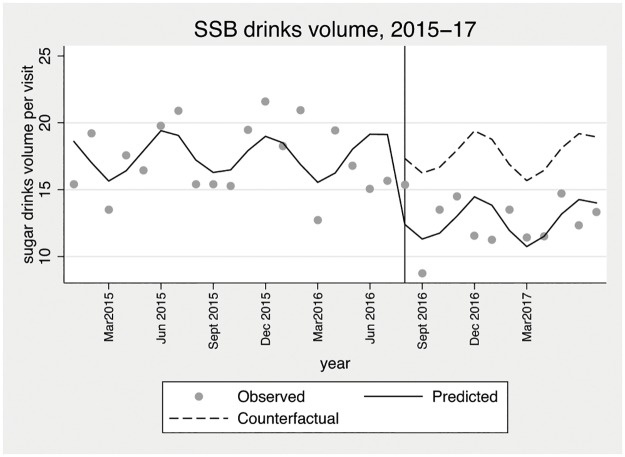
Monthly predicted sugar drinks volume (ml) per attendance comparing post-tax estimation with counterfactual prediction.

Table 3 summarises the results of the interrupted time series analysis of affected venues (see model 4 Tables B-H in S1 File). The interrupted time series analysis estimated a 31% (95% CI 4%, 59%) reduction in sales of SBB per attendance in the year since the policy was introduced. The estimated reduction in volume of SSB was similar, suggesting that customers were not switching purchases to larger volume SSB drinks to get better value for money. Fig 2 illustrates the gap between the post-tax estimated volume of sugar drinks consumed by customers and the predicted counterfactual with seasonal adjustment. The predicted sales rates are based on the seasonally adjusted fixed effects regression model (model 4), and the counterfactual illustrates this estimation in the absence of the intervention dummy term. There is no indication from Fig 2 that the effectiveness of the policy varied over the post-tax period included in this study. However, after only 12 months follow-up we have limited evidence on the long-term effectiveness of the policy. Detailed output for the interrupted time series analysis, including analyses by venue, are reported in S1 File. Overall the results were robust to removal of rainfall, seasonal adjustment, inclusion of school holiday dummy variables, and mixed effects model. Additional sensitivity analyses investigated whether the policy increased in effectiveness over time. The analysis revealed a possible time trend in the reduction in SSB sales per attendance but the result was not statistically significant (results not reported).

**Table 3:** Interrupted time series model 4 results: Estimated change in cold drinks sales and SSB volume (ml) per attendance before and after policy after adjusting for seasonality and rainfall.

	All cold drinks	SSB	Non-SSB	Non-SSB (excl. Juice and water)	Water	Fruit juice	SSB volume (ml)
Estimated sales per attendance before policy	0.100	0.043	0.057	0.026	0.019	0.012	15.785
Estimated sales per attendance after policy	0.095	0.030	0.066	0.033	0.020	0.012	11.451
Percentage change in sales per attendance	-4.70%	-31.44%*	15.83%*	26.52%*	6.88%	5.96%	-27.46%*

*P<0.05,

**P<0.01,

***P<0.001

The analysis identified an increase in sales per attendance for non-SSBs after the policy was introduced. There was a 16% (95% CI 1%, 30%) increase in sales of non-SSB per attendance (artificially sweetened sodas, energy drinks, juices, and water) in the post-policy period. Sales per attendance of water and low sugar fruit juice reported small non-significant changes in sales per attendance suggesting no change in healthy beverage consumption. Nevertheless, artificially sweetened carbonated drinks (excluding water and low sugar fruit juices) were highly responsive to the policy leading to a 27% (95% CI 6%, 47%) increase in sales per attendance. The impact of the policy on non-SSB options were highly variable across venues with almost no substitution observed for Concord and ICE Sheffield (comprising lower socioeconomic status and younger attendees respectively) but much higher in Ponds Forge in the city centre and Hillsborough suggesting that the response to the policy and impact on revenues are likely to be highly dependent on the population demographics (see Tables I-P in S1 File).

The log-log price elasticity of demand analysis identified that a 1% increase in price alongside health promotion leads to a 3.8% (95% CI 3.1% 4.4%) decrease in demand for SSB’s (Table 4). This demonstrates that demand for SSB in leisure venues were highly elastic and statistically significant. The analysis was also broken down product type. There is substantial variability in price elasticity between product types. Post mix drinks had the lowest price elasticity whilst juice from concentrate and energy drinks had very high price elasticities.

**Table 4:** Price elasticity of demand for all sugar sweetened beverages.

	SSB drinks at all venues by product type					
	All SSBs	Cans	Bottles	Post Mix (soda on draught)	Sweetened juice from concentrate	High caffeine energy
Intercept (*β*_0_)	6·008 (0.194)***	3·944 (0.396)***	6·344 (0.277)***	6·996 (0.379)***	4·832 (0.522)***	7·839 (0.950) ***
Own price elasticity of demand (*β*_1_)	-3.753 (0.328)***	-2.550 (0.695)***	-2.263 (0.433)***	-1.271 (0.619)*	-8.060 (0.861)***	-6·133 (1.417)***
Attendances at venue per month	0·009 (0.003)**	0·004 (0·010)	0·014 (0.004)**	0·003 (0·005)	0·020 (0.009)*	-0·007 (0·010)
Seasonal adjustment: Fourier term (1)	-0·107 (0·037)**	-0·180 (0·101)	-0·045 (0·047)	-0·024 (0·063)	-0·259 (0·110)*	-0·068 (0·088)
Seasonal adjustment: Fourier term (2)	-0·035 (0·038)	0·044 (0·103)	0·053 (0·048)	-0·046 (0·069)	-0·248 (0·118)*	-0·095 (0·089)
Seasonal adjustment: Fourier term (3)	0·032 (0·032)	-0·095 (0·091)	-0·031 (0·048)	0·040 (0·066)	0·182 (0·113)	0·118 (0.104)
Seasonal adjustment: Fourier term (4)	-0·025 (0·037)	-0.057 (0·091)	-0·075 (0·049)	0·015 (0·063)	0·101 (0·107)	-0·157 (0·086)

*P<0.05,

**P<0.01,

***P<0.001

Analysis of venues not affected by the SSB policy found no statistically significant changes in demand for SSB or non-SSB at these venues at the time of the policy introduction (See S1 File).

## Discussion

This study examines the changes in purchases of SSB at leisure centres over one year after a 20p price increase and promotion campaign was introduced. The average volume of SSBs purchased monthly was 31% lower compared with expected purchases in the absence of a price increase and health promotions policy. Purchases of non-SSBs were 16% higher after the price increase, may be indicative of substitution to artificially sweetened carbonated drinks rather than water or juice. Furthermore, our analyses show that demand for SSB in leisure centre venues is highly elastic. The overall and substitution effects varied between venues and product types, suggesting that consumers of drinks specifically marketed for children were more likely to reduce consumption and switch to alternatives. The very large price elasticity for these products may suggest that the policy is more effective for young people and, more specifically, when parents are purchasing drinks for their children. The changes in non-SSB sales per attendance in two venues suggest that substitution to artificially sweetened beverages may vary with visitor profile. One of these venues attracts a younger population (ICE Sheffield) and the other serves a more socioeconomically deprived area of the city (Concord).

### Comparison with other literature

This analysis demonstrates that the leisure centre pricing and promotions policy has been more effective in changing purchasing behaviour than observed in other studies. Previous policies from Mexico and a restaurant chain estimated decreases in sales from 9–12%,[6–7] whereas this study estimates a 30% reduction in sales of SSBs. Using data from the Living Costs and Food Survey in the UK, Briggs et al. estimate a 1% price increase leads to 0.92% decrease for concentrated sugar sweetened drinks and 0.81% decrease for non-concentrated drinks.[14] In another study of New Zealand the own price elasticity for SSBs varied from decreases of 0.14% to 3.47% per 1% price increase depending on income quintiles.[15] In a study from the US demand decreased by 1.03% for regular carbonated sugar sweetened drinks and 2.36% for sports/energy drinks per 1% increase in price.[16] In a study from Chile demand decreased by1.37%.[17] In contrast, our study found a 1% increase in price as part of a health promotion leads to a 3.8% decrease in demand. The large effect sizes observed in this study may be due to two factors. Firstly, the price increases were accompanied by staff training to draw customers attention to price differences, poster promotions, stickers identifying affected products and ensuring non-SSB are at eye-level. The analysis indicates that the combination of price rises and promotional policy changes are effective. A randomised controlled trial of vending machines interventions found that product replacements and promotions were more effective in encouraging healthy purchases than pricing alone. [18] Secondly, it has not been possible to evaluate how the policy has affected purchasing behaviour outside of these venues. It is possible that some customers delayed the purchase of SSBs and visited other retail outlets to avoid paying the higher price. This explanation is less likely to apply to customers choosing to substitute for non-SSB products. Given that we observed a large increase in non-SSB consumption and overall sales did not decrease significantly, we believe that there would be limited delayed purchases of SSB products outside the venues which might mitigate the effectiveness of the policy.

The results of this study should be reviewed in the context of the introduction of a national levy on SSB products in the UK in April 2018, which highlights that price increases will most likely lead to substitution to non-sugar alternatives. This study presents the first evaluation of a public health campaign within community leisure centres, which provides a unique setting to deliver public health policy. Leisure centres are part a complex system impacting on health and obesity through provision of physical activity facilities and broader health campaigns. We find that price increases, combined with poster displays, re-location of diet products and staff promotion, in this setting were more impactful than similar studies of pricing policy alone. This highlights the importance of modifications to the local food provision setting to enhance pricing policies and responds to calls for greater focus on systems approaches to public health interventions modifying multiple factors influencing SSB consumption. [19]

### Strengths and limitations

The policy has been evaluated in leisure centre venues across Sheffield serving a broad social spectrum. The results have shown that catering policies in leisure centres throughout the UK and internationally can be implemented to modify consumer behaviour and encourage more healthy drink choices. SCT attracts a high proportion of customers from lower socioeconomic groups due to the location of venues, suggesting that the policy can be effective in more hard to reach groups. Given the breadth of facilities on offer, the study population includes representation from wide age ranges, gender mix and BME groups. As such we believe that the results can be generalisable to other public catering facilities. The results suggest that adoption of price and health promotion policies on SSB products is an effective way to support public health efforts to reduce sugar consumption. This finding may encourage other leisure centres and public catering facilities to adopt similar health promotion policies.

This analysis retrospectively evaluated the policy and as such was not designed to establish causality in a controlled setting. Other changes are occurring concurrent with the tax, including anticipation of a national tax on SSB, health campaigns about sugar sweetened beverages, and anti-obesity programs may contribute to the demand for SSB in the leisure centre venues. The study did not identify any pre-existing trends in SSB in the pre-tax period. From our analysis of venues not affected by the policy it is clear that a similar reduction in SSBs were not observed in the post-policy period. The affected and unaffected venues were not randomly allocated, which means it is not possible to conclusively state that the changes observed are solely due to the new policy.

It was not possible to distinguish the effects of the price increase from the health promotional policies. In this study we have assessed the impact of the all measures implemented by SCT, but we cannot conclusively assess the additional benefits of the staff training and promotion in addition to the price changes. In future prospective policy evaluation designs it would be preferable to randomise venues to different components of the policy and estimate the incremental effectiveness of adding health promotion to a price increase in leisure centres.

The analysis utilises routinely collected data by SCT rather than data collected for the purposes of this study. While sales data are accurately recorded, attendance data relies on estimation methods to account for the number of parents/carers accompanying children for lessons, number of customers using booked sports facilities and spectators at un-ticketed events. We rely on the methods developed over time by SCT for accounting purposes believe these represent good approximations of the number of attendances each month. Furthermore, the methods are consistent over the study period.

Further research is needed to understand how customers viewed the policy and whether the policy affected purchasing behaviour outside of the venues. This study was not able to directly investigate how the policy was received by visitors and what factors motivated drink choices to reduce consumption of SSBs and switch to alternative products. Furthermore, a prospective randomised evaluation would strengthen future investigation of food and drinks policies to ensure that changes in behaviour are not due to other external factors.

## Conclusions

This study details the impact of a SSB pricing and promotion strategy in leisure centre venues and the findings are relevant for other leisure centres and catering facilities in public spaces. This analysis has useful lessons on how pricing and promotion can be used to promote healthy dietary behaviour changes in public places. Firstly, the price rises in leisure centres were effective in reducing purchases of high sugar beverages. Secondly, visual and verbal promotions of the policy at retail points and by staff are likely to have enhanced the effectiveness of the policy when the results are compared with other policy evaluations. Thirdly, the price elasticity of demand is highly variable across products, with the greatest reductions in child-friendly products, suggesting that parent’s purchases of food and drink for children may be particularly susceptible to these campaigns. Fourthly, the evaluation identified a large increase in sales of artificially sweetened beverages, suggesting customers are willing to substitute with diet products.

## Supporting information

S1 File (DOCX)

S2 File (XLSX)
