# Supplementary material for: The impact of a local sugar sweetened beverage health promotion and price increase on sales in public leisure centre facilities
Source: PLoS One. 2018 May 30;13(5):e0194637. doi: 10.1371/journal.pone.0194637 (PMC5976158; doi:10.1371/journal.pone.0194637)
Supplement: S1 File — (DOCX) [file pone.0194637.s001.docx]

**The impact of a local sugar sweetened drinks price increase on sales in leisure centre facilities**

**Supplementary Appendix**

# 1. Background: The Intervention

The package of changes made at leisure centre venues was developed in brainstorming meeting amongst some key managers including the CEO of Sheffield City Trusts. The response of stakeholders and focus group participants was overwhelmingly positive, with many customers advocating SCT to play a bigger role in promoting healthy lifestyles. The package of changes included price increases alongside other promotional ideas because multiple nudges were believed to be more effective in behaviour change than price increase alone. The policy was implemented in leisure centres rather than entertainment venues because high sugar drinks are purchased by a higher proportion of children than in entertainment venues. Targeting childhood obesity was a priority in designing the policy. All relevant aspects of the policy were adopted in all venues. For example, venues with only vending machines were not eligible for staff training, but affected products were given stickers. After consulting with customers the policy was designed with the following features.

The leisure venues cater for a wide range of people. SCT proactively provide leisure facilities to reach the most hard to reach groups and are successful at engaging people from BME backgrounds, people with disabilities and have a representative gender split across their usage.

Typically the venues have a high proportion of use from surrounding postcodes/geographical areas. The ‘community venues’ are incredibly successful at reaching into some of the most deprived areas in Sheffield – in particular Concord, Springs and Heeley attract people from areas with well recognised health inequalities. Other venues see a more city-wide spread, attracting people from across the city to visit for leisure experience.

SCT delivers a huge range of activities from children’s coached lessons to Exercise Memberships, thousands of fitness classes, golf, swimming and ice skating. The leisure centres cater for all ages and tend to attract a good spread of people across all ages.

1. Price increase. A 20p price increase on all drinks with 5mg sugar per 100ml was chosen rather than a 10p increase to increase the impact of the policy. The 5mg threshold was chosen in line with government recommendations of 5mg sugar per 100ml as criteria for levy, and exclude milk and fruit drink due to nutritional value.

2. Promotion of the policy within affected centres

a. Modified layout in drinks vending machines so that ‘healthier’ options are positioned at eye level

b. Tall posters positioned close to venue entrances with low sugar health promotion message.

c. Stickers on affected products indicating high sugar content.

3. Media publicity. Coverage of the policy in local print and radio media.

4. Staff training. Catering staff at cafes received training on the policy change, including the rationale for the policy to communicate to customers. Staff were encouraged to highlight the price difference to customers at the point of purchase, and sugar content to allow customers to change their purchase to a lower cost item.

The types of products affected by the pricing strategy are detailed in Table A, including details of the drinks volume.

Table A: Product types and prices before and after pricing strategy

| Product Type | Point of sale | Venues | Drinks Volume | Price July 2015 | Price August 2016 |
| --- | --- | --- | --- | --- | --- |
| Carbonated Soft drink Can | Vending | PFO, CSC, SPR, ICE, EISS | 330ml | £0.80 | £1.00 |
| Carbonated Soft drink Bottle | Cafe/vending | HLC, PFO, CSC, SPR, ICE, EISS, HEE | 500ml | £1.40 | £1.60 |
| Carbonated Soft Drink Post mix* cup | Cafe | HLC, PFO, ICE | 280ml | £1.20 | £1.40 |
| Sweetened juice from concentrate | Cafe/vending | HLC, PFO, SPR, ICE, EISS, HEE | 200ml | £1.00 | £1.20 |
| High caffeine energy drink | Cafe/vending | HLC, CSC, ICE, EISS | 500ml | £1.80 | £2.00 |
| Carbonated mixer drinks | Cafe | ICE | 200ml | £1.20 | £1.40 |
| Carbonated mixer drinks | Cafe | ICE | 125ml | £1.00 | £1.20 |
| * Soda on draught | | | | | |

# 2. Results: Interrupted time series – All affected venues

Tables B-H report the parameter estimates and standard errors for all drinks types. Models 1-4 are estimated using fixed effects models, and model 6 presents the results with a mixed effects model.

Table B: Regression output for total sales per person

| Total sales per person regression results | | | | | |
| --- | --- | --- | --- | --- | --- |
|  | Model 1 | Model 2 | Model 3 | Model 4 | Model 5 |
|  | Unadjusted regression | Binary seasonal adjustment | Smooth seasonal adjustment | Smooth seasonal adjustment and rainfall | Mixed effects model |
| Intercept ($\beta_{0}$) | 0.1002 (0.002)*** | 0.0950 (0.002)*** | 0.0990 (0.002)*** | 0.1043 (0.005)*** | 0.1031 (0.032)** |
| Policy dummy ($\beta_{1}$) | -0.0048 (0.005) | -0.0047 (0.005) | -0.0036 (0.005) | -0.0047 (0.005) | -0.0047 (0.005) |
| School Holidays |  | 0.0122 (0.007) |  |  |  |
| Fourier term (1) |  |  | -0.0005 (0.003) | -0.0006 (0.003) | -0.0006 (0.003) |
| Fourier term (2) |  |  | 0.0013 (0.002) | 0.001 (0.002) | 0.001 (0.003) |
| Fourier term (3) |  |  | -0.0090 (0.006) | -0.008 (0.006) | -0.008 (0.003) |
| Fourier term (4) |  |  | 0.0053 (0.005) | 0.006 (0.005) | 0.006 (0.003) |
| Rainfall |  |  |  | -0.0001 (0.000) | -0.0001 (0.00) |

Table C: Regression output for SSB sales per person

| SSB sales per person regression results | | | | | |
| --- | --- | --- | --- | --- | --- |
|  | Model 1 | Model 2 | Model 3 | Model 4 | Model 5 |
|  | Unadjusted regression | Binary seasonal adjustment | Smooth seasonal adjustment | Smooth seasonal adjustment and rainfall | Mixed effects model |
| Intercept ($\beta_{0}$) | 0.043 (0.002)*** | 0.042 (0.001)*** | 0.043 (0.002)*** | 0.046 (0.003)*** | 0.046 (0.018) |
| Policy dummy ($\beta_{1}$) | -0.013 (0.005)* | -0.013 (0.005)* | -0.013 (0.005)* | -0.014 (0.005)* | -0.014 (0.005)** |
| School Holidays |  | 0.004 (0.003) |  |  |  |
| Fourier term (1) |  |  | 0.000 (0.001) | 0.0003 (0.001) | 0.0002 (0.001) |
| Fourier term (2) |  |  | 0.000 (0.001) | -0.0003 (0.001) | -0.0004 (0.001)* |
| Fourier term (3) |  |  | -0.003 (0.002)* | -0.003 (0.002) | -0.003 (0.002)* |
| Fourier term (4) |  |  | 0.003 (0.003) | 0.003 (0.003) | 0.003 (0.002) |
| Rainfall |  |  |  | -0.000 (0.000) | -0.000 (0.000) |

Table D: Regression output for SSB volume per person

| SSB volume per person regression results | | | | | |
| --- | --- | --- | --- | --- | --- |
|  | Model 1 | Model 2 | Model 3 | Model 4 | Model 5 |
|  | Unadjusted regression | Binary seasonal adjustment | Smooth seasonal adjustment | Smooth seasonal adjustment and rainfall | Mixed effects model |
| Intercept ($\beta_{0}$) | 15.784 (0.645)*** | 15.237 (0.250*** | 15.681 (0.574)*** | 16.31 (0.843)*** | 16.100 (6.098) |
| Policy dummy ($\beta_{1}$) | -4.302 (1.643)* | -4.294 (1.042)* | -4.198 (1.586)* | -4.334 (1.633)* | -4.309 (0.504)** |
| School Holidays |  | 1.292 (1.042) |  |  |  |
| Fourier term (1) |  |  | -0.151 (0.405) | -0.168 (0.400) | -0.187 (0.504) |
| Fourier term (2) |  |  | -0.267 (0.357) | -0.290 (0.364) | -0.306 (0.488) |
| Fourier term (3) |  |  | -0.825 (0.664) | -0.752 (0.674) | -0.767 (0.503) |
| Fourier term (4) |  |  | 1.228 (0.938) | 1.355 (0.964) | 1.361 (0.011) |
| Rainfall |  |  |  | -0.009 (0.006) | -0.008 (0.013) |

Table E: Regression output for non-SSB sales per person

| Non-SSB sales per person regression results | | | | | |
| --- | --- | --- | --- | --- | --- |
|  | Model 1 | Model 2 | Model 3 | Model 4 | Model 5 |
|  | Unadjusted regression | Binary seasonal adjustment | Smooth seasonal adjustment | Smooth seasonal adjustment and rainfall | Mixed effects model |
| Intercept ($\beta_{0}$) | 0.0568 (0.001)*** | 0.0534 (0.002)*** | 0.0561 (0.001)*** | 0.058 (0.003)*** | 0.057 (0.016)** |
| Policy dummy ($\beta_{1}$) | 0.0087 (0.003)* | 0.0087 (0.003)* | 0.0087 (0.003)* | 0.0089 (0.003)* | 0.0089 (0.003)** |
| School Holidays |  | 0.0079 (0.002) |  |  |  |
| Fourier term (1) |  |  | -0.0008 (0.002) | -0.0009 (0.002) | -00009 (0.002) |
| Fourier term (2) |  |  | 0.0015 (0.001) | 0.0014 (0.001) | 0.0015 (0.002) |
| Fourier term (3) |  |  | -0.0058 (0.004) | -0.0056 (0.004) | -0.0056 (0.002) |
| Fourier term (4) |  |  | 0.0027 (0.002) | 0.0031 (0.003) | 0.0031 (0.002) |
| Rainfall |  |  |  | -0.0000 (0.000) | -0.000 (0.000) |

Table F: Regression output for non-SSB sales (excl. water and juice) per person

| Non-SSB (excl. juice and water) sales per person regression results | | | | | |
| --- | --- | --- | --- | --- | --- |
|  | Model 1 | Model 2 | Model 3 | Model 4 | Model 5 |
|  | Unadjusted regression | Binary seasonal adjustment | Smooth seasonal adjustment | Smooth seasonal adjustment and rainfall | Mixed effects model |
| Intercept ($\beta_{0}$) | 0.027 (0.001)*** | 0.025 (0.002)*** | 0.026 (0.001)*** | 0.026 (0.002)*** | 0.026 (0.010)* |
| Policy dummy ($\beta_{1}$) | 0.007 (0.002)* | 0.007 (0.002)* | 0.007 (0.002)* | 0.007 (0.002)* | 0.007 (0.002)** |
| School Holidays |  | 0.005 (0.003) |  |  |  |
| Fourier term (1) |  |  | -0.001 (0.001) | -0.001 (0.001) | -0.001 (0.001) |
| Fourier term (2) |  |  | 0.000 (0.000) | -0.0003 (0.000) | -0.0003 (0.001) |
| Fourier term (3) |  |  | -0.003 (0.002) | -0.003 (0.002) | -0.003 (0.001) |
| Fourier term (4) |  |  | 0.002 (0.002) | 0.002 (0.002) | 0.002 (0.001) |
| Rainfall |  |  |  | 0.000 (0.000) | 0.000 (0.000) |

Table G: Regression output for juice sales per person

| Juice sales per person regression results | | | | | |
| --- | --- | --- | --- | --- | --- |
|  | Model 1 | Model 2 | Model 3 | Model 4 | Model 5 |
|  | Unadjusted regression | Binary seasonal adjustment | Smooth seasonal adjustment | Smooth seasonal adjustment and rainfall | Mixed effects model |
| Intercept ($\beta_{0}$) | 0.012 (0.0004)*** | 0.011 (0.008)*** | 0.012 (0.0004)*** | 0.012 (0.001)*** | 0.012 (0.003)** |
| Policy dummy ($\beta_{1}$) | 0.0005 (0.001) | 0.0005 (0.001) | 0.0007 (0.001) | 0.001 (0.001) | 0.001 (0.001) |
| School Holidays |  | 0.002 (0.001)* |  |  |  |
| Fourier term (1) |  |  | 0.001 (0.001) | 0.000 (0.001) | 0.001 (0.001) |
| Fourier term (2) |  |  | 0.001 (0.000)* | 0.001 (0.001) | 0.001 (0.001) |
| Fourier term (3) |  |  | -0.001 (0.001) | -0.001 (0.001) | -0.001 (0.001) |
| Fourier term (4) |  |  | 0.000 (0.000) | -0.000 (0.001) | -0.000 (0.001) |
| Rainfall |  |  |  | -0.000 (0.000) | 0.000 (0.000) |

Table H: Regression output for water sales per person

| Water sales per person regression results | | | | | |
| --- | --- | --- | --- | --- | --- |
|  | Model 1 | Model 2 | Model 3 | Model 4 | Model 5 |
|  | Unadjusted regression | Binary seasonal adjustment | Smooth seasonal adjustment | Smooth seasonal adjustment and rainfall | Mixed effects model |
| Intercept ($\beta_{0}$) | 0.0185 (0.001)*** | 0.0179 (0.001)*** | 0.0183 (0.000)*** | 0.0202 (0.001)*** | 0.019 (0.005)*** |
| Policy dummy ($\beta_{1}$) | 0.0015 (0.001) | 0.0015 (0.001) | 0.0017 (0.001) | 0.0013 (0.001) | 0.001 (0.001) |
| School Holidays |  | 0.0014 (0.001) |  |  |  |
| Fourier term (1) |  |  | -0.0003 (0.001) | -0.0004 (0.001) | -0.000 (0.001) |
| Fourier term (2) |  |  | 0.0007 (0.000) | 0.0006 (0.000) | 0.001 (0.001) |
| Fourier term (3) |  |  | -0.0016 (0.001) | -0.0014 (0.001) | -0.001 (0.001)* |
| Fourier term (4) |  |  | 0.0010 (0.000) | 0.0014 (0.001) | 0.001 (0.001)* |
| Rainfall |  |  |  | -0.0000 (0.000)* | -0.000 (0.000) |

# 2. Interrupted time-series regression by venue

Tables I-P reports the estimated percentages of sales per attendance before the policy and impact of tax policy on sales by venue for vending only centres. The tables report the sales per attendance estimated from the regression analysis with and without the policy dummy term.

Table I: Estimated change in cold drinks sales per attendance before and after policy in Hillsborough adjusting for seasonal variation and rainfall

|  | All soft drinks sales | High sugar drinks | Low sugar drinks | Low sugar soft drinks (excl. Juice and water) | Fruit juice | Water |
| --- | --- | --- | --- | --- | --- | --- |
| Estimated sales per attendance before policy | 0.079 | 0.034 | 0.045 | 0.017 | 0.014 | 0.014 |
| Estimated sales per attendance after policy | 0.064 | 0.019 | 0.045 | 0.019 | 0.012 | 0.013 |
| Percentage change | -18.93% | -42.95%*** | -0.87% | 13.53% | -16.24% | -2.49% |

Table J: Estimated change in cold drinks sales per attendance before and after policy in Ponds Forge adjusting for seasonal variation and rainfall

|  | **All cold drinks** | **SSB** | **Non-SSB** | **Non-SSB (excl. Juice and water)** | **Fruit juice** | **Water** |
| --- | --- | --- | --- | --- | --- | --- |
| Estimated sales per attendance before policy | 0.145 | 0.052 | 0.092 | 0.034 | 0.020 | 0.038 |
| Estimated sales per attendance after policy | 0.153 | 0.038 | 0.115 | 0.051 | 0.019 | 0.044 |
| Percentage change | 5.78% | -27.44%** | 24.70%* | 50.87%*** | -5.30% | 17.27% |

Table K: Estimated change in cold drinks sales per attendance before and after policy in Concord

|  | All soft drinks sales | High sugar drinks | Low sugar drinks | Low sugar soft drinks (excl. Juice and water) | Fruit juice | Water |
| --- | --- | --- | --- | --- | --- | --- |
| Estimated sales per attendance before policy | 0.049 | 0.015 | 0.034 | 0.014 | 0.008 | 0.012 |
| Estimated sales per attendance after policy | 0.042 | 0.009 | 0.033 | 0.015 | 0.007 | 0.012 |
| Percentage change | -14.63% | -39.04%* | -4.03% | 10.14% | -11.01% | -15.49% |

Table L: Estimated change in cold drinks sales per attendance before and after policy in Springs

|  | All soft drinks sales | High sugar drinks | Low sugar drinks | Low sugar soft drinks (excl. Juice and water) | Fruit juice | Water |
| --- | --- | --- | --- | --- | --- | --- |
| Estimated sales per attendance before policy | 0.020 | 0.010 | 0.010 | 0.003 | 0.002 | 0.006 |
| Estimated sales per attendance after policy | 0.020 | 0.003 | 0.017 | 0.008 | 0.003 | 0.006 |
| Percentage change | 1.31% | -65.20% | 62.74% | 151.48% | 78.59% | 11.38% |

Table M: Estimated change in cold drinks sales per attendance before and after policy in ICE Sheffield

|  | All soft drinks sales | High sugar drinks | Low sugar drinks | Low sugar soft drinks (excl. Juice and water) | Fruit juice | Water |
| --- | --- | --- | --- | --- | --- | --- |
| Estimated sales per attendance before policy | 0.272 | 0.150 | 0.122 | 0.079 | 0.017 | 0.026 |
| Estimated sales per attendance after policy | 0.244 | 0.109 | 0.134 | 0.089 | 0.018 | 0.027 |
| Percentage change | -10.53% | -27.02%** | 9.71% | 12.95% | 4.50% | 3.40% |

Table N: Estimated change in cold drinks sales per attendance before and after policy in English Institute for Sport

|  | All soft drinks sales | High sugar drinks | Low sugar drinks | Low sugar soft drinks (excl. Juice and water) | Fruit juice | Water |
| --- | --- | --- | --- | --- | --- | --- |
| Estimated sales per attendance before policy | 0.108 | 0.033 | 0.075 | 0.032 | 0.015 | 0.029 |
| Estimated sales per attendance after policy | 0.108 | 0.033 | 0.075 | 0.032 | 0.015 | 0.029 |
| Difference (coefficient $\beta_{1}$) | 3.30% | -37.36%* | 21.02% | 26.68%** | 40.30% | 5.21% |

Table O: Estimated change in cold drinks sales per attendance before and after policy in Heeley

|  | All soft drinks sales | High sugar drinks | Low sugar drinks | Low sugar soft drinks (excl. Juice and water) | Fruit juice | Water |
| --- | --- | --- | --- | --- | --- | --- |
| Estimated sales per attendance before policy | 0.011 | 0.004 | 0.007 | 0.002 | 0.002 | 0.003 |
| Estimated sales per attendance after policy | 0.017 | 0.002 | 0.014 | 0.006 | 0.004 | 0.005 |
| Difference (coefficient $\beta_{1}$) | 51.31% | -41.92% | 97.47% | 234.69% | 66.09% | 45.74% |

Table P: Estimated change in cold drinks sales per attendance before and after policy in vending only centres

|  | All soft drinks sales | High sugar drinks | Low sugar drinks | Low sugar soft drinks (excl. Juice and water) | Fruit juice | Water |
| --- | --- | --- | --- | --- | --- | --- |
| Estimated sales per attendance before policy | 0.027 | 0.010 | 0.018 | 0.006 | 0.004 | 0.007 |
| Estimated sales per attendance after policy | 0.027 | 0.005 | 0.022 | 0.010 | 0.005 | 0.007 |
| Difference (coefficient $\beta_{1}$) | -1.33% | -46.93%*** | 23.39%* | 53.92%** | 15.10% | 1.09% |

# 3. Interrupted time-series analysis with non-policy venues

Table Q reports the estimated sales per attendee for unaffected venues over the study period with a dummy variable indicating the policy introduction.

Table Q: Estimated change in cold drinks in unaffected venues only

|  | All soft drinks sales | High sugar drinks | Low sugar drinks | Low sugar soft drinks (excl. Juice and water) | Fruit juice | Water |
| --- | --- | --- | --- | --- | --- | --- |
| Intercept ($\beta_{0}$) | 0.198 (0.050)*** | 0.073 (0.018)*** | 0.122 (0.032)*** | 0.085 (0.015) *** | 0.014 (0.009) *** | 0.024 (0.012) *** |
| Policy dummy ($\beta_{1}$)* | -0.030 (0.034) | -0.020 (0.013) | -0.011 (0.022) | -0.003 (0.011) | -0.001 (0.006) | -0.007 (0.009) |
| Fourier term (1) | -0.008 (0.025) | 0.002 (0.009) | -0.010 (0.016) | -0.006 (0.008) | -0.003 (0.004) | -0.001 (0.006) |
| Fourier term (2) | -0.045 (0.025) | -0.018 (0.009) | -0.027 (0.016) | -0.013 (0.009) | -0.006 (0.004) | -0.008 (0.006) |
| Fourier term (3) | 0.037 (0.024) (0.024) | 0.015 (0.009) | 0.022 (0.015) | 0.005 (0.007) | 0.008 (0.004) | 0.008 (0.008) |
| Fourier term (4) | 0.019 (0.023) | 0.011 (0.009) | 0.008 (0.015) | 0.002 (0.007) | 0.003 (0.004) | 0.003 (0.006) |
| Rainfall | 0.001 (0.001) | 0.000 (0.000) | -0.000 (0.000) | 0.000 (0.000) | 0.000 (0.000) | 0.000 (0.000) |
| *Policy dummy included from August 2016; | | | | | | |
